# Evaluation of a 12-hole clock model for improving bronchoscopic skills in simulated normal and difficult airways among anesthesia residents: A randomized controlled study

**DOI:** 10.1097/MD.0000000000038510

**Published:** 2024-06-07

**Authors:** Zhiqiang Zhou, Kaiwen Zhang, Xu Zhao, Yingjie Hu, Yuqin He, Li Wan, Wenlong Yao

**Affiliations:** a Department of Anesthesiology, Hubei Key Laboratory of Geriatric Anesthesia and Perioperative Brain Health, Wuhan Clinical Research Center for Geriatric Anesthesia, Tongji Hospital, Tongji Medical College, Huazhong University of Science and Technology, Wuhan, China; b Department of Radiology, Tongji Hospital, Tongji Medical College, Huazhong University of Science and Technology, Wuhan, China.

**Keywords:** airway, bronchoscopy, cognitive load, intubation, simulation training

## Abstract

**Background:**

Simulation-based training is used to improve fiberoptic bronchoscopic skills for novices. We developed a nonanatomical task trainer (named 12-hole clock model) that focused on training manipulation of bronchoscopes. The aim of this study was to evaluate the training effect of this model on bronchoscopic skills and learning interests in simulated normal and difficult airways among anesthesia residents.

**Methods:**

Forty-three anesthesia residents without experience in bronchoscopic intubation were randomly divided into control (n = 22) and intervention groups (n = 21). All participants received standard multimedia learning and a baseline test using a normal airway manikin. Then, the control and intervention groups engaged in 60 minutes of training via a traditional airway manikin or the clock model, respectively. After training, the participants completed bronchoscopic performance assessments in simulated normal and difficult airways, as well as an electronic questionnaire related to the course.

**Results:**

During training, the total hands-on time of bronchoscopic practice recorded by trainees’ themselves was longer in the intervention group than in the control group (1568 ± 478 seconds vs 497 ± 172 s, *P* < .0001). Posttraining, the time required to visualize the carina in simulated normal airways was longer in the intervention group than in the control group (22.0 [18.0, 29.0] vs 14.0 [10.8, 18.3], *P* < .0001), while it was shorter for simulated difficult airways (24.0 [16.0, 32.0] s vs 27.0 [21.0, 35.5] s, *P* = .0425). The survey results indicated that confidence in bronchoscopic intubation increased in both groups, without significant differences in satisfaction, acceptance, or perceived difficulty between the groups. However, the interest ratings were higher in the intervention group than in the control group.

**Conclusions:**

The 12-hole clock model is a simple and feasible method for improving bronchoscopic skills and promoting interest among trainees.

**Trial registration:**

NCT05327842 at Clinicaltrials.gov.

## 1. Introduction

In the field of anesthesia, flexible fibreoptic bronchoscopes (FOB) or video bronchoscopes are often used in situations with difficult airways or to confirm the position of the double-lumen tube. Although awake bronchoscopic intubation is still considered the first choice for anticipated difficult intubation,^[1]^ the increased popularity of videolaryngoscopy has dramatically decreased the incidence of difficult airways, and the use of bronchoscopic intubation has become rare.^[2,3]^ Thus, residents may have limited opportunities to learn bronchoscopic intubation techniques, which are more difficult than videolaryngoscopy techniques and are associated with a steeper learning curve.^[4]^

Simulation-based training is vital for preventing ethical and safety issues when attempting to teach the basic clinical skills to novices.^[5,6]^ Previous studies have demonstrated that both simple models and high-fidelity simulators are effective in improving bronchoscopic manipulation skills.^[7]^ Virtual reality simulators can be used to present realistic clinical scenarios,^[8,9]^ which can stimulate interest and improve learning efficiency.^[10]^ However, such approaches are expensive. Whether the expense of acquisition of virtual reality hardware and software is needed is an open question. Bronchoscopic manipulation skills may decay over time, necessitating periodic practice to maintain an acceptable level of proficiency.^[11,12]^ A simple, portable, and much less expensive model may be valuable in many hospitals.

**Figure 1.**
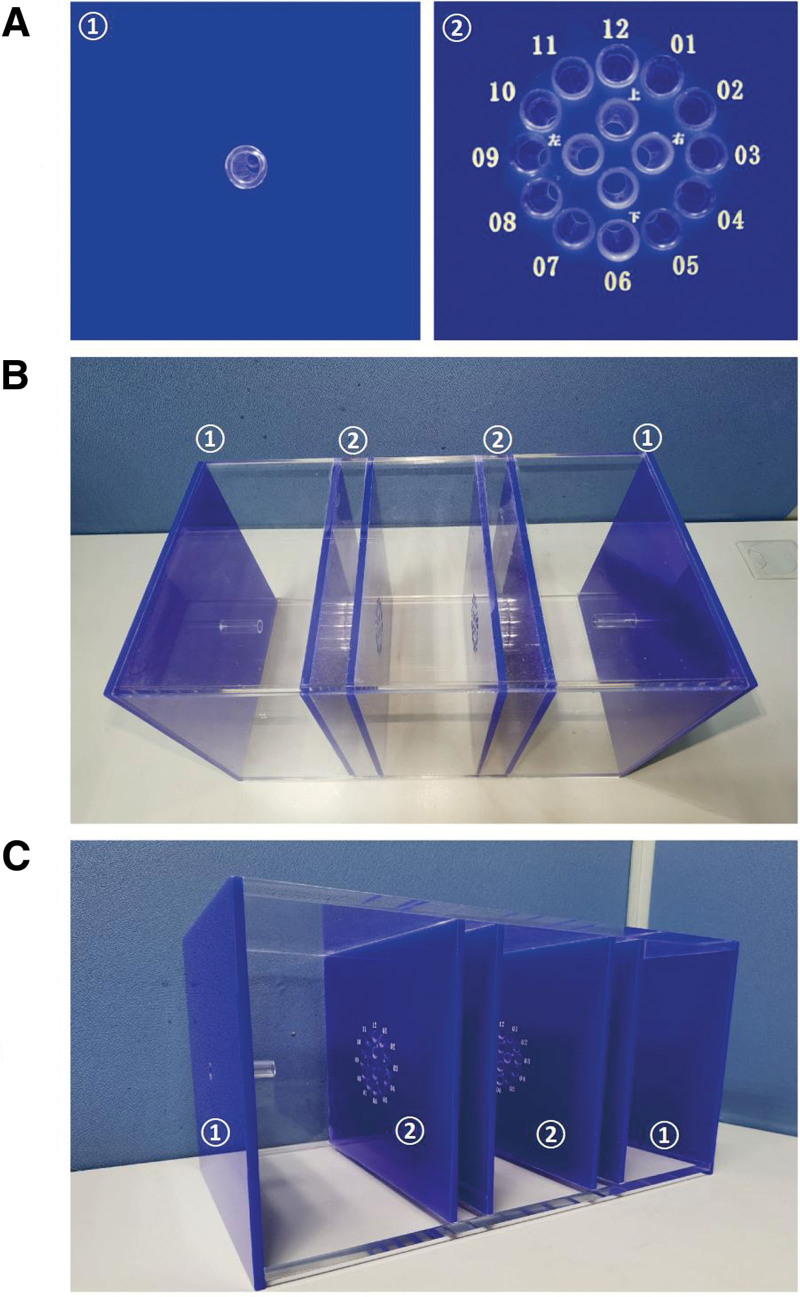
The 12-hole clock model. (A) ① 1st layer, ② 2nd layer; (B) vertical view; (C) lateral view. In (B, C), the 2-ended assembly enables simultaneous use by 2 trainees and participation in head-to-head competition to further enhance motivation to use the simulator.

Naik et al^[13]^ designed a simple yet effective wooden box model for training residents in bronchoscopic manipulation skills that can be transferred to the operating room. Based on the original model and cognitive load theory, we modified a 12-hole clock model (ZL201921518855.8, China) in which manipulation of the FOB was correlated with the familiar concept of the clock to promote retention in long-term memory and aid trainees in adjusting the direction and angle of the FOB (shown in Fig. 1). It was a nonanatomical task trainer that focused on training manipulation of FOB, separated from learning the anatomy. In a development phase, we observed this model may increase the difficulty of training, but the process may be more interesting and engaging for trainees, which may further aid in improving eye-hand coordination and refining key bronchoscopic manipulation skills.

In this study, we aimed to investigate the training effect of a 12-hole clock model on bronchoscopic skills in simulated normal and difficult airways among a cohort of anesthesia residents by comparison with a traditional airway manikin.

## 2. Methods

### 2.1. Participants

**Figure 2.**
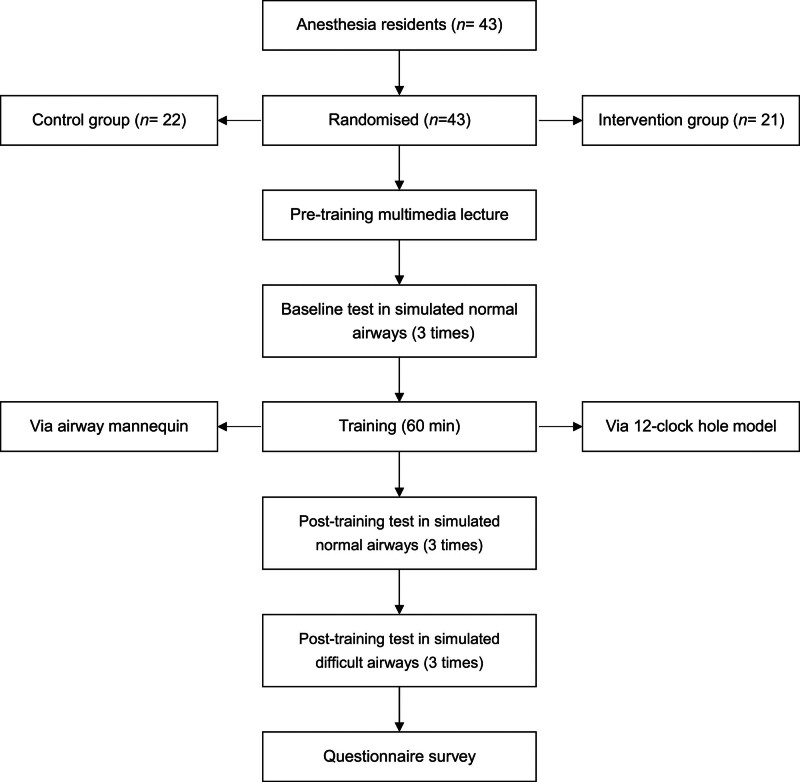
Flow chart of the training process.

This study was performed in Department of Anesthesiology, Tongji Hospital, Huazhong University of Science and Technology, Wuhan, China. The study protocol was approved by the Ethics Committee of Tongji Hospital, Tongji Medical College, Huazhong University of Science and Technology (TJ-IRB20220515) and registered at Clinicaltrials.gov (Ref. NCT05327842). Written informed consent was obtained from all the participants. Fifty anesthesia residents in their 1st or 2nd year of training at our institution were enrolled, all of whom had general experience with airway management. Demographic data, including age, sex, and clinical experience, were collected. After excluding 7 patients with clinical experience in fibreoptic manipulation, 43 participants were allocated to the control group (n = 22) or the intervention group (n = 21) using a computer-generated random number list. A flowchart of the training process was shown in Figure 2.

### 2.2. Training course

Before training, all participants underwent standard multimedia education regarding basic FOB skills and key points of bronchoscopic intubation. Thereafter, the control and intervention groups engaged in 60 minutes of training involving a traditional airway manikin with normal airways (Laerdal Airway Management Trainer, Laerdal Medical) or a 12-hole clock model (Fig. 1; ZL201921518855.8), respectively. In the control group, the participants were required to manipulate FOB (UE Medical Corp., outer diameter 5.8 mm) pass from the mouth to the carina. In the intervention group, the participants were required to manipulate FOB sequentially pass from the 1st to 12th hole. During training, 2 experienced anesthesiologists provided real-time guidance and direct feedback on performance. Within 60 minutes of training, at least of 20 attempts were required in the control group and 3 attempts in the intervention group. The participants could choose to continue practice according to their wishes until the time was up. For each attempt, the participants recorded the hands-on time of bronchoscopic practice by themselves with a timer. The total hands-on time of bronchoscopic practice was calculated by summing the times of all attempts for each participant.

### 2.3. The 12-hole clock model

As shown in Figure 1, there are 2 layers in the model. After passing through the hole in middle of the 1st layer, the FOB reaches a 2nd layer in which 12 holes are arranged in a circle, as in a standard 12-hour clock. It allows the trainee to manipulate the FOB pass through a particular target hole. For example, when the target hole is located at the 3 o’clock position, the operator can push the control lever of the FOB downward to direct the tip of the device toward the 12 o’clock position, following which it can be rotated 90° clockwise to reach the target. In our training, the trainee was required to manipulate FOB sequentially pass from the 1st to 12th hole.

### 2.4. Pretraining and posttraining tests

All participants underwent a pretraining test in a simulated normal airway and posttraining tests in simulated normal and difficult airways. Tests were monitored by an independent mentor blinded to the group allocation. In the control group, the same manikin was used for training and the normal airway test. A difficult airway test involved a simulated case of ankylosing spondylitis. The cervical spine and jaw of the manikin were fixed to simulate neck rigidity, limited mouth opening, and a short thyromental distance. Each test was performed in triplicate. The time required to manipulate the FOB from passage the incisor to achieving visualization of the carina was recorded. A time longer than 120 seconds was considered a failure.

### 2.5. Questionnaire

Following training, participants completed an electronic questionnaire related to the training course, which included items related to confidence in bronchoscopic intubation before and after training, as well as items related to satisfaction, acceptance, interest, and difficulty. Satisfaction was classified into 3 levels (high, moderate, and low). For the other items, participants provided ratings on a scale of 1 to 100, with 1 indicating an extreme lack of confidence and 100 indicating extreme confidence.

### 2.6. Outcomes

The primary outcome of the current study was the time required to visualize the carina in the simulated difficult airway via FOB manipulation. Secondary outcomes included success rates and times required for carina visualization in the simulated normal airway, the total hands-on time of bronchoscopic practice, and self-ratings related to use of the FOB and the training course.

### 2.7. Statistical analysis

IBM SPSS Statistics for Windows, version 19.0 (IBM Corp., Armonk, NY, USA) was used for statistical analysis. For continuous variables, we expressed normally distributed data as means with standard deviations (SDs) and used independent *t* tests to conduct between-group comparisons, while non-normally distributed data were expressed as medians with interquartile ranges (IQRs) and Mann–Whitney *U* tests for comparisons. We used chi-square and Fisher exact tests, as appropriate, to compare categorical data. Statistical significance was set at *P* < .05.

Sample size was calculated according to procedure time. Every participant performed 3 attempts in simulated difficult airways. To detect a 10 seconds difference in procedure time with a standard deviation of 18 seconds based on previous reports, an a priori power analysis revealed that a group size of 17 was needed to detect a difference with a power of 0.8 and an α level of 0.05. We recruited more than 20 participants per group to allow for dropouts.

## 3. Results

### 3.1. Demographic data

**Figure 3.**
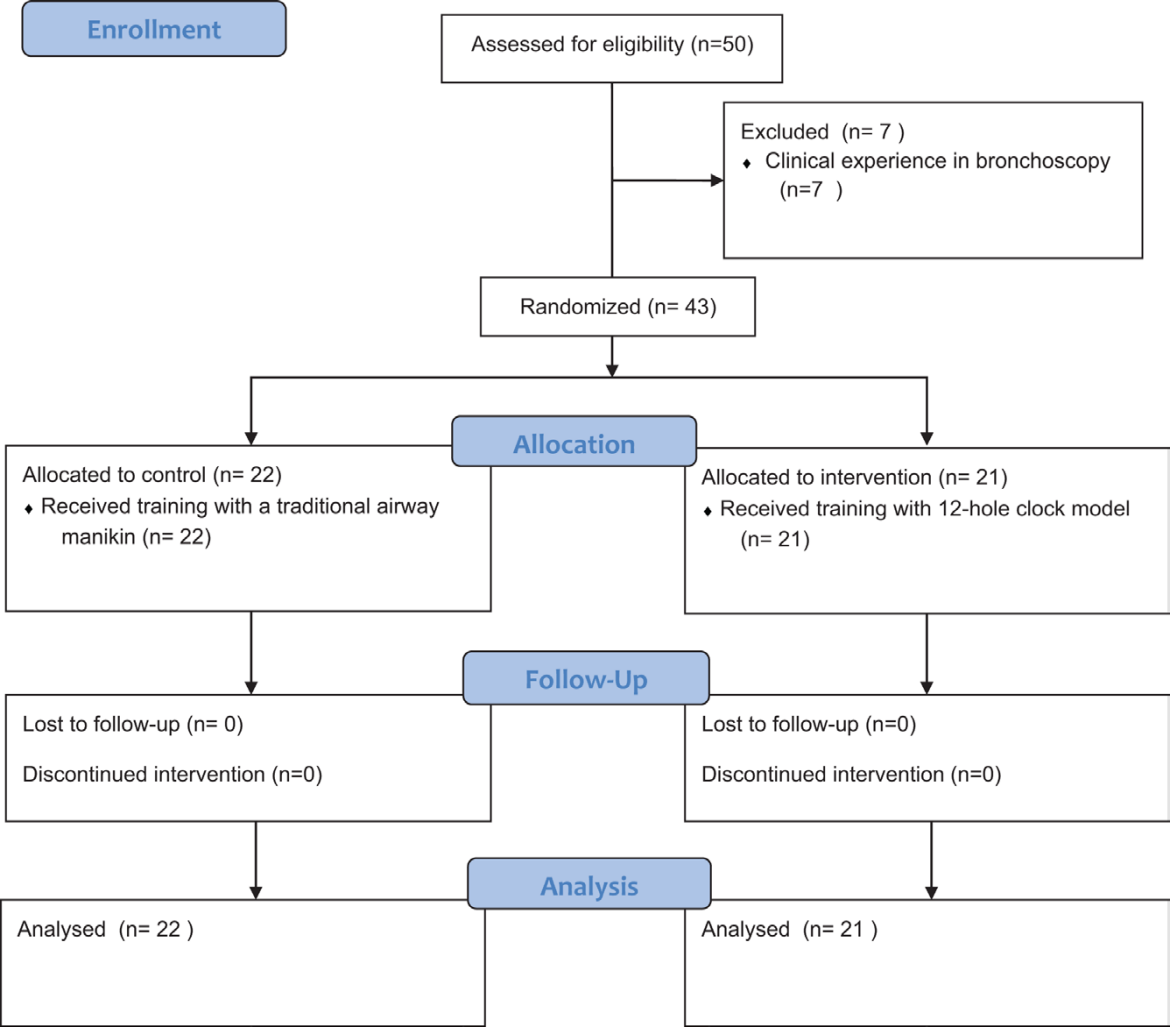
CONSORT flow diagram.

The CONSORT flow diagram was presented in Figure 3. Seven participants with bronchoscopic experience were excluded. As shown in Table 1, there were no significant differences in sex, age, or clinical experience between the 2 groups. Although all participants engaged in 60 minutes of training, the total hands-on time of bronchoscopic practice was longer in the intervention group than in the control group (1568 ± 478 seconds vs 497 ± 172 s, *P* < .0001).

**Table 1:** Demographic characteristics and training time.

	Control group (n = 22)	Intervention group (n = 21)	*P*
Sex, male/female	5/17	7/14	.438
Age (yr)	26.7 ± 3.2	27.0 ± 2.8	.770
Clinical years	1.1 ± 1.3	1.6 ± 1.4	.263
Total hands-on time of bronchoscopic practice (s)	497 ± 172	1568 ± 478	<.0001

### 3.2. Posttraining test

As shown in Table 2, the posttraining times for visualization of the carina with FOB were significantly shorter than the pretraining times in both groups. After training, the time to visualization of the carina in simulated normal airways was longer in the intervention group than in the control group (22.0 [18.0, 29.0] vs 14.0 [10.8, 18.3], *P* < .0001). However, in difficult airways, the time to visualization of the carina was shorter in the intervention group than in the control group (24.0 [16.0, 32.0] s vs 27.0 [21.0, 35.5] s, *P* = .0425).

**Table 2:** Bronchoscopic performance in simulated normal and difficult airways.

	Control group (n = 66)	Intervention group (n = 63)	*P*
Pretraining test in simulated normal airways			
Time to visualization of the carina (s)	53.0 (31.0, 86.8)	62.0 (39.0, 106.0)	.2287
Success rate, n (%)	57 (86.4%)	50 (79.4%)	.291
Posttraining test in simulated normal airways			
Time to visualization of the carina (s)	14.0 (10.8, 18.3)*	22.0 (18.0, 29.0)*	<.0001
Success rate, n (%)	66 (100%)	63 (100%)	1.000
Posttraining test in simulated difficult airways			
Time to visualization of the carina (s)	27.0 (21.0, 35.5)	24.0 (16.0, 32.0)	.0425
Success rate, n (%)	65 (99.2%)	63 (100%)	1.000

Data are expressed as the median (interquartile range) or n (%).

* *P* < .0001, compared with pretraining test.

### 3.3. Self-evaluation

The Cronbach α for the electronic questionnaire was 0.809. As shown in Table 3, paired-samples *t* tests revealed that, when compared with before-training results, confidence in bronchoscopic intubation significantly increased in both groups after training, although there was no significant difference between the 2 groups (unpaired *t* test). The satisfaction, acceptance, and difficulty ratings were comparable between the 2 groups. However, ratings of interest were higher in the intervention group than in the control group (85.6 ± 12.4 vs 75.4 ± 17.9, *P* = .036).

**Table 3:** Questionnaire results.

	Control group (n = 22)	Intervention group (n = 21)	*P*
Confidence in FOI before training (T1)	40.6 ± 25.2	31.0 ± 18.1	.159
Confidence in FOI after training (T2)	78.7 ± 12.1*	80.0 ± 13.8*	.749
Satisfaction with training course (high/moderate/low)	18/4/0	16/5/0	.721
Acceptance of training	91.6 ± 10.5	92.3 ± 11.0	.833
Interest in training	75.4 ± 17.9	85.6 ± 12.4	.036
Difficulty of training	78.3 ± 17.2	86.0 ± 12.2	.098

Data are expressed as the median (interquartile range) or n.

FOI = fibreoptic intubation.

* *P* < .0001, compared with T1.

## 4. Discussion

Low-fidelity training methods for FOB manipulation include wooden box models,^[13]^ nonanatomic endoscopic dexterity training systems,^[6]^ and 3D-printed bronchoscopy simulators.^[14]^ High-fidelity methods include pig lungs and virtual reality bronchoscopy simulators.^[15]^ Previously, Chandra et al^[7]^ reported no difference in skill transfer to intraoperative patient care between low- and high-fidelity training models. Although Jiang et al^[8]^ reported that training based on a GI-Bronch Mentor virtual reality simulation was more efficient than manikin-based training among novices in FOB, they observed no significant differences in procedure time or global rating scale scores after 25 training sessions. These results indicate that low-fidelity models represent a viable alternative for FOB training in anesthesia, which is particularly advantageous for training programs with budgetary constraints.

The cognitive load theory has important implications in medical education.^[16]^ It assumes that working memory can process only a limited number of informational elements at any given time,^[17]^ with types of cognitive load classified into 3 categories: extraneous, intrinsic, and germane. When cognitive overload occurs during a task, learning and performance are impaired. The germane load, a learning-relevant load considered to complement extraneous and intrinsic loads,^[18]^ is associated with the mental work a person needs to perform to build organized knowledge structures and automate that knowledge during the learning process. In addition, during FOB manipulation, clinicians must ensure rapid and accurate control of the device tip in a 3-dimensional space when attempting to reach the target point.^[19]^ Based on these theories, we incorporated the familiar concept and designed a 12-hole clock model. Using this model, sequential manipulation of the FOB from the 1st to 12th hole allows for comprehensive understanding and training regarding adjustments to the FOB in all directions. As the exterior box of this model is transparent, trainees can also directly observe the movement of the FOB from outside the visual field to obtain a more thorough understanding of the FOB dynamics. Repeated and deliberate practice using this model can help trainees solidify these representations in their long-term memory.

In this study, we found that success rates and times for visualization of the carina in simulated normal airways improved in both groups following training. Although participants trained using the 12-hole clock model took longer to visualize the carina in the normal airway test than those trained using a traditional airway manikin, they took less time in the difficult airway test than the control group. This may be because the training was performed using the same manikin and airway conditions as the control group. In addition, manipulating FOB to visualize the carina is relatively easy in manikins with normal airways.^[20]^ Thus, the participants in the control group were more familiar with the pharyngeal structure of the model, which may explain their relatively better performance in the normal airway test.

Simulator-based training and assessment of airway skills should provide a reliable measure of performance in clinical situations with a range of airway problems and degrees of difficulty.^[10,21]^ The main indication for FOB is the need to manage difficult airways. Therefore, we modified the traditional training manikin to simulate a difficult airway in a patient with ankylosing spondylitis, which is a typical clinical scenario. The conditions simulated in the difficult airway model included cervical hyperflexion, a narrow pharyngeal cavity, and obstruction of the glottis by the epiglottis, which required trainees to adjust the tip of the FOB so that it passed over the epiglottis to view the glottis. Given that the tip must be finely adjusted in difficult airways, FOB manipulation is more challenging in difficult airways than in normal airways, particularly when glottic exposure or tracheal intubation is difficult. In our study, participants trained using the 12-hole clock model required less time to visualize the carina in difficult simulated airways than those trained using the traditional manikin, suggesting that the 12-hole clock model is relatively more effective for training. However, given that the total hands-on time of fiberoptic practice was longer in the intervention group than in the control group, this relative improvement in the training effect may have been related to the training intensity of the model itself or the longer overall training time. Further studies are required to explore the influence of the total training time on FOB proficiency.

Learning bronchoscopic intubation skills requires a high cognitive load for novices.^[22]^ Although training is often performed using manikins with normal airways to aid novices in acquiring basic skills for FOB manipulation, trainees can reach a learning plateau within a short time, indicating an insufficient intrinsic cognitive load. Furthermore, simple task may not sufficiently engage or motivate the trainees. The 12-hole clock model utilized in the current study increases intrinsic cognitive load by allowing for adjustment of the FOB in different directions. Using a familiar concept (i.e., a standard, 12-h clock face) may aid in promoting retention of knowledge and skills in long-term memory. Therefore, this training method manages the working memory load within acceptable limits by increasing the germane load to stimulate interest and improve the performance.

Our results demonstrated that during training, the total hands-on time of bronchoscopic practice was longer in the intervention group than in the control group. One possible reason is that the training content for the control group was relatively monotonous. After several training sessions, participants may have reached a learning plateau, leading to a lack of interest in continuing the training. In contrast, participants in the intervention group were required to perform multiple tasks by manipulating FOB to different target holes, which forced them to spend more time in practicing FOB. The posttraining survey showed significant improvements in confidence were observed after training in both groups, indicating that both the normal airway and 12-clock hole models were effective. Moreover, there was no difference in the ratings of satisfaction or acceptance between the 2 groups, indicating that the total cognitive load was acceptable to all participants. However, there was a statistically significant difference in the interest in the training course between the intervention and control groups. Although ratings of difficulty were higher in the intervention group than in the control group, the difference was not statistically significant, consistent with cognitive load theory, and indicative of a greater interest in learning when using the 12-clock hole model. Therefore, despite the increased challenges of training using this model, it is easy to understand and may be more interesting for trainees.

The current study had some limitations, especially since the training course did not involve the placement of a tracheal tube under FOB guidance. Although simulator training enables trainees to master the core skills of FOB and obtain adequate visualization of the glottis and carina, the complete process for FOB-guided intubation requires not only exposure of the glottis, but also railroading of the tube over the FOB.^[23]^ Additionally, we did not evaluate whether the training effect could be transferred to clinical practice by testing FOB-guided intubation in patients undergoing surgery. Thus, participants are likely to require further simulation or clinical training. Finally, as we only evaluated objective proficiency based on performance time, further studies are required to determine the effect of the 12-hole clock model on global rating scale scores.

## 5. Conclusions

In summary, the current findings highlight the 12-hole clock model as a simple and feasible method for bronchoscopic training, which may stimulate interest and improve bronchoscopic skills among trainees. The model used in this study was no more than US$ 100. Therefore, it is highly affordable for most hospitals and is suitable for large-scale institutional use.

## Author contributions

**Conceptualization:** Zhiqiang Zhou, Wenlong Yao.

**Formal analysis:** Zhiqiang Zhou, Xu Zhao.

**Investigation:** Zhiqiang Zhou, Wenlong Yao.

**Writing – original draft:** Zhiqiang Zhou, Xu Zhao.

**Data curation:** Kaiwen Zhang, Yingjie Hu, Yuqin He.

**Supervision:** Li Wan, Wenlong Yao.

**Writing – review & editing:** Li Wan, Wenlong Yao.

**Funding acquisition:** Wenlong Yao.
